# Age-Dependent Effect of Transcranial Alternating Current Stimulation on Motor Skill Consolidation

**DOI:** 10.3389/fnagi.2020.00025

**Published:** 2020-02-06

**Authors:** Shane Fresnoza, Monica Christova, Lara Bieler, Christof Körner, Ulrike Zimmer, Eugen Gallasch, Anja Ischebeck

**Affiliations:** ^1^Institute of Psychology University of Graz, Graz, Austria; ^2^BioTechMed, Graz, Austria; ^3^Otto Loewi Research Center, Division of Physiology, Medical University of Graz, Graz, Austria; ^4^Institute of Physiotherapy, University of Applied Sciences FH-JOANNEUM, Graz, Austria; ^5^Faculty of Human Sciences, Medical School Hamburg (MSH), Hamburg, Germany

**Keywords:** transcranial alternating current stimulation, motor learning, alpha frequency, age-dependent, skill consolidation

## Abstract

Transcranial alternating current stimulation (tACS) is the application of subthreshold, sinusoidal current to modulate ongoing brain rhythms related to sensory, motor and cognitive processes. Electrophysiological studies suggested that the effect of tACS applied at an alpha frequency (8–12 Hz) was state-dependent. The effects of tACS, that is, an increase in parieto-occipital electroencephalography (EEG) alpha power and magnetoencephalography (MEG) phase coherence, was only observed when the eyes were open (low alpha power) and not when the eyes were closed (high alpha power). This state-dependency of the effects of alpha tACS might extend to the aging brain characterized by general slowing and decrease in spectral power of the alpha rhythm. We additionally hypothesized that tACS will influence the motor cortex, which is involved in motor skill learning and consolidation. A group of young and old healthy adults performed a serial reaction time task (SRTT) with their right hand before and after the tACS stimulation. Each participant underwent three sessions of stimulation: sham, stimulation applied at the individual participant’s alpha peak frequency or individual alpha peak frequency (iAPF; α-tACS) and stimulation with iAPF plus 2 Hz (α2-tACS) to the left motor cortex for 10 min (1.5 mA). We measured the effect of stimulation on general motor skill (GMS) and sequence-specific skill (SS) consolidation. We found that α-tACS and α2-tACS improved GMS and SS consolidation in the old group. In contrast, α-tACS minimally improved GMS consolidation but impaired SS consolidation in the young group. On the other hand, α2-tACS was detrimental to the consolidation of both skills in the young group. Our results suggest that individuals with aberrant alpha rhythm such as the elderly could benefit more from tACS stimulation, whereas for young healthy individuals with intact alpha rhythm the stimulation could be detrimental.

## Introduction

Aging is defined as a persistent decline in the age-specific fitness components of an organism due to internal physiological deterioration (Galloway, 1993). In humans, one of the hallmarks of aging-associated deterioration is a deficit in motor performance which includes coordination, balance, and gait difficulties, as well as slowing and increased variability of movement (Seidler et al., 2010). The age-related motor deficits also extend to the learning of new motor skills and the modification of previously learned skills (King et al., 2013). The acquisition and consolidation of implicit motor sequence skills are reported to be preserved and impaired, respectively, while the reverse is observed for visuomotor adaptation skills (King et al., 2013). These motor behavioral changes in the elderly were suggested to be causally linked to the age-related structural deterioration in brain areas responsible for movement planning and execution. For instance, decreased gray matter volume in the cerebellum, caudate nucleus, prefrontal, parietal and sensorimotor cortices, as well as white matter deterioration in the corpus callosum, corticospinal tract and cerebellum are consistent findings in older adults when compared to young adults (Good et al., 2001; Salat et al., 2004; Raz et al., 2005; Ota et al., 2006; Sullivan et al., 2010). Furthermore, a significant decline in dopamine, acetylcholine, serotonin and norepinephrine-mediated neurotransmission in older adults have also been directly linked to a deficit in motor performance including skill learning (Seidler et al., 2010).

More recently, research has focused on the age-related changes in the oscillatory activity in the alpha frequency band and its significance for the neural control of movement during physiological aging. Several studies involving elderly participants identified voluntary movement planning and execution related electroencephalography (EEG) changes within the alpha frequencies in the parieto-occipital area (“classical” or posterior alpha rhythm) and the sensorimotor cortex (motor-cortical alpha, “mu,” or Rolandic rhythm). For instance, the absence of motor-cortical alpha and limited low beta lateralization (motor-related amplitude asymmetries or MRAA) in internal motor preparation was concomitant with slowed reaction time and suggested less efficient cerebral processes subserving free movement selection in older adults, which may indicate a reduced capacity for internally driven action with age (Deiber et al., 2014). Similarly, aging influenced the patterns of event-related desynchronization/synchronization (ERD/ERS). There were reports of larger alpha and beta ERD amplitude, increased spatial diffusion of motor-cortical alpha ERD over the parietal and frontal regions, and lengthening of motor-cortical alpha ERD duration during finger movements in the elderly subjects compared with young subjects (Derambure et al., 1993; Labyt et al., 2003; Deiber et al., 2014; Mary et al., 2015; Quandt et al., 2016). Cued finger movements, pinches, and the whole hand grip task also elicited a widespread spatial distribution and more uniform flat curve of alpha power decrease in the elderly compared to young participants in sensorimotor areas, which were linked to age-related changes in the neural coding of skilled motor behavior (Quandt et al., 2016). The post-movement rebound of magnetoencephalography (MEG) motor-cortical alpha and beta activity in the sensorimotor cortex, which was suggested to reflect plasticity changes, is also impaired in the aging brain (Mary et al., 2015). Furthermore, there is less increase in motor-cortical alpha power than in older adults during the inhibition of learned movements, which is believed to be due to a deficit in local inhibitory mechanisms within the sensorimotor cortices (Bönstrup et al., 2015). On the other hand, motor cortical potentials that preceded freely-executed voluntary finger or hand movements (lateralized readiness potential or LRP) were reported to increase in aged individuals. Specifically, an increase in the amplitude of response-locked LRPs was indicative of a slowed motor response (Feve et al., 1991; Yordanova et al., 2004; Roggeveen et al., 2007; Cespón et al., 2013).

Aging also affects alpha oscillatory activity in other brain regions. With regard to the posterior alpha rhythm (8–13 Hz), there was a marked reduction in amplitude, slowing of spontaneous oscillation, and declined reactivity (eye-opening) which correlated with global cognitive performance in the elderly (Babiloni et al., 2006; Ishii et al., 2017; Knyazeva et al., 2018). Older adults also showed reduced connectivity in the upper alpha band compared to young adults (Kikuchi et al., 2000; Vysata et al., 2014; Scally et al., 2018). Other changes due to aging include a significant alpha increase in frontal regions, mainly over the prefrontal cortex (PFC), which has also been observed in early Alzheimer’s disease patients (Kolev et al., 2002; Ishii et al., 2017). The shift of alpha from posterior to frontal regions has been associated with a compensatory mechanism. Older adults need to activate the PFC even to accomplish very easy tasks, whereas younger adults do not have to draw on this cortical resource (Mattay et al., 2002; Berchicci et al., 2012). The evidence so far, thus suggests a causal link between impaired alpha activity and cognitive and motor impairment in healthy aging, as well as a link to pathological conditions such as Alzheimer’s disease (Ishii et al., 2017; Koelewijn et al., 2017). It is therefore timely to develop interventions that can modulate the alpha rhythm in the elderly.

Transcranial alternating current stimulation (tACS) can directly modulate specific cortical oscillations and with it possibly cognitive and motor functions (Antal and Paulus, 2013). The exact neurophysiological mechanism behind the effects of tACS remains unclear. In animal models, it was shown that neuronal entrainment, that is, synchronization of endogenous (cortical) oscillations with the extrinsically applied rhythmic current, was possible (Fröhlich and McCormick, 2010; Ali et al., 2013; Schmidt et al., 2014; Aspart et al., 2018; Toloza et al., 2018). In healthy human subjects (simultaneous tACS-EEG recording) and epilepsy patients (simultaneous direct cortical stimulation and electrocorticography (ECoG) recording), alpha tACS led to an increase in alpha power (Helfrich et al., 2014; Alagapan et al., 2016). It has also been shown that the alpha power increase lasted minutes until hours after the stimulation, which was associated with entrainment echoes or spike-timing-dependent plasticity (Zaehle et al., 2010; Neuling et al., 2013; Strüber et al., 2015; Vossen et al., 2015; Alagapan et al., 2016; Kasten et al., 2016). Behaviorally, tACS at individual alpha peak frequency (iAPF) was shown to increase target detection and mental rotation performance of young healthy participants, as well as improved inhibitory abilities in elderly participants during a working memory task (Helfrich et al., 2014; Kasten and Herrmann, 2017; Borghini et al., 2018). Interestingly, the observed increase in alpha power at the parieto-occipital cortex was found to be state-dependent. An increase in EEG/ECoG alpha power and MEG phase coherence was only observed when the participant’s alpha power was low (eye-open condition) and not when the participant’s alpha power was high (eyes-closed condition; Neuling et al., 2013; Alagapan et al., 2016; Ruhnau et al., 2016). Considering that alpha activity is usually reduced in the elderly (Ishii et al., 2017), we may speculate that alpha tACS will be more beneficial for this age group.

In this study, we investigated the state-dependent effect of alpha tACS on motor consolidation. A group of healthy young and old participants performed a serial reaction time task (SRTT) with their right hand before and after tACS stimulation. During tACS, the current was delivered to the left motor cortex at the iAPF in one session (α-tACS) and above the iAPF (iAPF + 2 Hz) in another session (α2-tACS). So far, this is the first study trying to find behavioral evidence of the state-dependent effect of tACS applied in the alpha band. Pioneering studies in healthy young participants suggest that the effect of tACS is also modulated by concurrent motor-cortical alpha activity. For instance, 10 Hz tACS increased the size of motor evoked potential (MEP) only during motor imagery and had no effect without it (Feurra et al., 2013). This can be explained by the state-dependency of tACS as motor-cortical alpha and beta bands recorded over the somatosensory and motor cortex desynchronize (power decrease) during this task (Nam et al., 2011; Kim and Lee, 2015; Galdo-Alvarez et al., 2016). It follows, that the effect of tACS on SRTT performance should be increased due to the reduced alpha activity during the task (Alagapan et al., 2016). Therefore, we hypothesized that α-tACS and α2-tACS stimulation of the motor cortex will improve the consolidation of motor skills in old participants more than in young participants.

## Materials and Methods

### Participants

Twenty healthy young adults between 19 and 30 years old (10 males; mean age 23.8 ± 3.90 years) and fifteen healthy older adults between 55 and 67 years old (six males; mean age 61.66 ± 3.71 years) participated in the study. The young group was composed of university students and the old group was composed of retirees with 14.06 ± 2.93 mean years of education. All participants had a normal or corrected-to-normal vision and were right-handed according to the Edinburgh Handedness Inventory (Oldfield, 1971). Exclusion criteria included any history of chronic medical or neuropsychiatric disorders (e.g., depression, epilepsy, and stroke), learning disability, brain injuries, intake of maintenance medications, and contraindications to tACS such as metallic or electrical implants in the body or the head (Poreisz et al., 2007). Individuals who were familiar with activities involving repeated sequential finger movements such as professional musicians or video game players, as well as those who had previous experience with the SRTT were also excluded. The study was approved by the Ethics Committee of the Medical University of Graz and all performed experimental procedures conformed to the principles of the Helsinki Declaration regarding human experimentation. All participants provided written informed consent prior to the experiment and received monetary compensation (60 Euros) for their participation in the study.

### Experimental Design and Procedures

The study was conducted in a single-blinded, randomized and sham-controlled design. Participants completed three randomized experimental sessions, two sessions with real tACS stimulation (applied at iAPF or “α-tACS” and iAPF + 2 Hz or “α2-tACS”) and one session with sham stimulation. To avoid carry-over effects, an interval of at least 1 week separated the experimental sessions. All experiments were carried out in the middle of the day (12:00–15:00) to ensure the highest alpha activity (Higuchi et al., 2001). The experiments were performed inside a dimly lit and sound-attenuated room. During the experiment, participants sat in a comfortable reclining chair with head and arm supports in front of a 19-inch computer monitor used to present the stimuli. Initially, the tACS electrodes were fixed underneath the EEG cap using rubber strips. In each session, a 5-min spontaneous resting-state EEG (eyes-open with central fixation) was recorded from three posterior electrodes to identify the participant’s iAPF before the stimulation. Participants were asked to relax and keep their eyes open during the measurements. The EEG data were immediately analyzed offline using a customized Matlab-based algorithm (Matlab R2016a, The MathWorks Inc., Natick, MA, USA) and the participant’s iAPF was identified from the Pz electrode. Participants were then asked to perform the SRTT as a baseline measure. After setting the individual stimulator frequency, the stimulation was started. To assess the impact of the stimulation on the early stages of motor skill consolidation, the participants performed the SRTT immediately after (0 min), 60 min and 120 min after stimulation. The EEG cap and tACS electrodes were removed during the first break. On average, each experimental session including the preparations and breaks lasted for 3 h.

### EEG Recording

The EEG recording was conducted using BrainAmp standard amplifiers with the recording software BrainVision Recorder (Brain Products GmbH, Gilching, Germany). Continuous EEG signals were collected from three Ag-AgCl electrodes (Cz, Pz, and Oz) embedded in an elastic cap housing 32 channels labeled in accord with an extended international 10-20 system (Easycap, Falk Minow, Munich, Germany). The ground electrode was located on the forehead (FPz electrode). For recording, the reference electrode was placed at the left mastoid. A second reference channel was placed at the right mastoid to enable an offline re-referencing to the linked mastoids. For monitoring eye movements, three EOG channels were used, two were placed on the outer canthi of the eyes and one was placed superior to the nasion. EEG and EOG signals were digitized at a sampling rate of 500 Hz and band-pass filtered between 0.1 Hz to 100 Hz. An additional 50 Hz notch filter was applied. Electrode impedances were maintained below 5 kΩ for the EEG recording and below 10 kΩ for the EOG recording.

### TACS Stimulation

tACS was delivered through a pair of saline-soaked (0.9%-NaCl) surface sponge electrodes connected to a battery-driven stimulator (ELDITH DC-stimulator, NeuroConn, Germany). The center of the first electrode (35 cm^2^, 0.04 mA/cm^2^ current density) was located underneath the C3 electrode of the EEG cap (10–20 EEG system) which corresponds to the location of the left primary motor cortex (left M1). This electrode was tangentially placed at a 45° angle relative to the central sulcus. A large second electrode (100 cm^2^, 0.01 mA/cm^2^ current density) was placed on the right contralateral supraorbital area (RO; Nitsche et al., 2007). The difference in the electrode sizes increased the focality of the stimulation since the current density was higher over the motor cortex than at the RO (Nitsche et al., 2007; Faria et al., 2011). Current flow modeling suggests that an adequate current can reach the motor cortex with a left M1-RO electrode montage without the influence of head fat distribution that may be different between young and old people (Miranda et al., 2006; Truong et al., 2013). The stimulation intensity was 1.5 mA (peak-to-peak current, no DC offset, no phase shift) and was applied for 10 min during the real tACS stimulation sessions. In one of the sessions, the current was delivered at the participant’s iAPF (α-tACS) determined from the Pz electrode. The stimulation parameters in this session were identical to the parameters we used in a recent study where we showed an increase in corticospinal excitability in both young and old individuals after α-tACS stimulation (Fresnoza et al., 2018). In another session, the current was delivered at the iAPF + 2 Hz (α2-tACS) in order to explore the frequency-specific effect of tACS on motor skill consolidation. To minimize the tingling skin sensation, the impedance during stimulation was maintained below 10 kΩ. In the sham stimulation session, the current was applied at α-tACS for only 30 s (with 10 s current ramping) and then switched off automatically without the participant’s awareness. This ensured that participants felt the same skin sensation in the sham condition as in the real stimulation conditions. In the present study, we used the iAPF determined from the posterior brain region as the stimulation frequency because of the significant role of the parietal cortex during motor preparation. The parietal cortex contributes to the temporal integration of sensory information into the movement sequence in order to ensure that each movement occurs after the successful completion of the preceding one (Catalan et al., 1998; Gongora et al., 2016). The sequence representation in the parietal cortex is then sent to the motor cortex for movement initiation (Yokoi et al., 2018). The posterior parietal cortex is also related to spatial goal-directed action planning and decisions of hand choice (Lindner et al., 2010; Oliveira et al., 2010; Petzschner and Krüger, 2012).

### Serial Reaction Time Task (SRTT)

**Figure 1:**
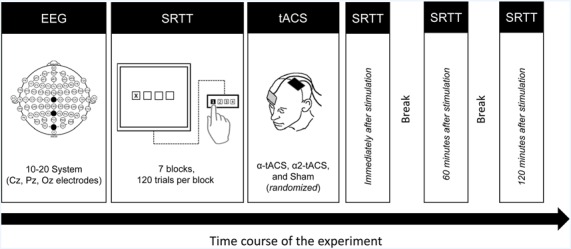
Experimental design. First, the participants underwent a 5-min resting-state EEG measurement to identify their individual alpha peak frequency (iAPF). Then they performed the SRTT task using their right hand. Immediately after the task, they received either α-tACS, α2-tACS or sham stimulation of the left motor cortex. They were retested immediately after, 60 min and 120 min after the stimulation. EEG, electroencephalography; SRTT, serial reaction time task; tACS, transcranial alternating current stimulation; α-tACS, tACS applied at iAPF; α2-tACS, tACS applied at iAPF + 2 Hz.

In the present study, we administered a modified version of the SRTT used in previous tDCS and tACS studies (Nitsche et al., 2003; Antal et al., 2008; Kuo et al., 2008; Pollok et al., 2015). Stimulus presentation and response recording were accomplished by using a program written in Psychopy (Psychology Software in Python, University of Nottingham; Peirce, 2009). A computer with a separate response box was used to record reaction times (RT) and error rate (ER). During the experiment, participants were seated in front of a computer screen. Their right-hand fingers rested on four horizontal buttons of a response box (index finger for button 1, middle finger for button 2, ring finger for button 3, and little finger for button 4; Figure 1). Each trial began with the presentation of four horizontally arranged square boxes of equal sizes in the center of the computer screen. The visual cue indicating the button (a cross that appeared in any of the four boxes) was presented in black on a white background. The participants were instructed to press a button of the response box that corresponded to the location of the cued box, as fast and as accurately as possible. Regardless of a correct or incorrect response, once a button was pressed, the cross disappeared and the trial ended. 500 ms later the cue appeared in a different box (Figure 1). One SRTT run was composed of seven blocks with 120 trials (button presses) each and took approximately 12–15 min to complete. Participants could take a break between blocks. In blocks 1 and 6, the appearance of the cross followed a pseudorandom or unpredictable order in which the cross was presented equally often in each position and never in the same position in two subsequent trials (random blocks). Blocks 2–5 and 7, were composed of 10 repetitions of the same 12-item button press sequence (1–2–1–4–2–3–4–1–3–2–4–3, sequential blocks). The participants were not informed about the existence of the repeating sequences. All participants performed four SRTT runs, one before stimulation (baseline) and three after stimulation (immediately after, 60 min and 120 min after stimulation). To test whether explicit knowledge of the sequences was acquired, the participants were asked after each run whether they had noticed a repeating sequence and if they could remember it. The RT for each trial is defined as the time difference (in milliseconds) between the onset of the cross (go signal) and the pressing of the correct button. On the other hand, the ER represents the ratio between the number of errors (wrong button presses and missing responses) within a block and the total number of trials on that block. The RTs and ERs before and after the stimulation were analyzed.

## Statistical Analysis

### EEG Data

The EEG data were preprocessed and analyzed offline using Brain Vision Analyzer software (version 2.01, Brain Products GmbH, Munich, Germany). First, data were offline re-referenced to a linked-mastoid reference. Then artifacts like eye blinks and other non-cerebral signals were removed using a thresholding method (WITHRESH function). Subsequently, the continuous data were epoched into 2,000 ms segments. Then, a Fast Fourier transformation (FFT) algorithm with a maximum resolution of 0.25 Hz (50% Hanning window) was used to calculate the power spectral density with a confidence interval boundary of 90% (Welch’s method). The iAPF was defined as the frequency where the maximum power was observed within the alpha range of 8–13 Hz (Klimesch, 1999).

### SRTT Data

The statistical analysis of the behavioral data (RT and ER) was performed using SPSS 22.0 software (IBM SPSS Statistics, IBM Corp., Armonk, NY, USA). From our raw RT data, we computed two scores to capture motor learning consolidation, the general motor skill (GMS) and sequence skill (SS) score (Walker et al., 2003). ER was calculated for each block of all the SRTT runs. The GMS score represents the speeding up of RTs across sequential blocks due to practice (Savic and Meier, 2016). Specifically, the GMS score is the mean RT difference between two sequential blocks and served as a measure of GMS learning and consolidation. A positive GMS score means that a participant performed faster on the second sequential block, which represents learning and consolidation. Conversely, a negative GMS score means that a participant performed slower on the second block. The SS score, on the other hand, indicates the increase in RT when switching from sequential blocks to random blocks which immediately followed (Nissen and Bullemer, 1987; Salthouse et al., 1999; Brown et al., 2009; Urry et al., 2018). Task routine is assumed to be equal in both blocks so that the RT difference represents implicit motor sequence learning (Pascual-Leone et al., 1994). In our task, the SS score represents the RT difference between a random block and the preceding sequential block and served as a measure of SS learning and consolidation. A positive SS score means that the RTs are longer in the random block and represents learning and consolidation. Conversely, a negative SS score means that the RTs in the random block were shorter than those in a sequential block.

### Skill Acquisition Stage

To evaluate motor skills acquisition before the stimulation in both groups, a GMS score was calculated from block 2 (sequential) and block 7 (sequential) while an SS score was calculated from block 6 (random) and block 5 (sequential) of the first SRTT run. Statistical comparison of the GMS and SS scores was performed using an independent sample *t*-test (paired, two-tailed).

### Skill Consolidation Stage

In order to measure the effect of tACS on the “off-line” consolidation of GMS, we calculated the in-between SRTT run GMS scores. Here, the GMS score represents the RT difference between block 7 of the first SRTT run and block 2 of the subsequent SRTT run. We used block 2 rather than block 1 of the next SRTT run because block 1 was random and the RT difference between block 7 and block 1 might underestimate the extent of GMS consolidation (Meier and Cock, 2014). Three GMS scores were determined: the RT difference before stimulation (block 7) and immediately after stimulation (block 2); immediately after (block 7) and 60 min after stimulation (block 2); 60 min after (block 7) and 120 min after stimulation (block 2). On the other hand, the effect of stimulation on the SS consolidation was assessed based on the SS scores calculated within the SRTT runs (RTs in block 6 minus the RTs in block 5). In total, we have four SS scores, one before and three after stimulation (immediately after, 60 min, and 120 min after stimulation).

The GMS scores, SS scores, and ERs were separately analyzed using linear mixed-effects modeling (LMM) with random-intercept. Linear models are robust alternatives to pure ANOVAs when dealing with unbalanced datasets like in our study (20 young and 15 old participants; Searle, 1988; Warton et al., 2016). The GMS and SS scores from incorrect trials and trials with RTs ±2 standard deviations (outliers) were excluded from the analysis. In the models, the GMS score, SS score, and ER served as the dependent variables and participants were included as a random factor. The between-subjects factor group (young vs. old) and the within-subjects factors stimulation (sham, α-tACS, and α2-tACS) and time (GMS score: 0 min, 60 min, and 120 min; SS score: baseline, 0 min, 60 min, and 120 min) were treated as fixed-effect covariates. For the GMS score, the score before the stimulation, calculated from two sequential blocks within the first SRTT run, was not included (GMS baseline score). Therefore, only the GMS scores (three) calculated between SRTT runs and SS scores (four) calculated within the SRTT runs using the raw RTs were entered into the models. In the separate model for the ER, blocks (1–7) were added as a within-subjects factor. Normal data distribution and homogeneity of variance test were conducted using Shapiro–Wilk and Levene’s test, respectively.

We performed a model selection procedure to determine the most parsimonious model for our data using a (forward) stepwise approach (Barr et al., 2013). We started with baseline models that only contained the random factor subject (to examine the individual variation in the dependent variable regardless of the other predictors) and then incrementally added the predictors (Singer and Willett, 2003). The within-subjects factors were then added to the model followed by the between-subjects factor group, as well as their respective interactions. By adding a factor to the model one-at-a-time, we were able to compare the Akaike Information Criterion (AIC) values that indicate model adequacy (Akaike, 1973). This method can determine overfitting in the model because it penalizes the likelihood function for having too many parameters. Model fit improvement or worsening was indicated by a 2-point decrease or increase in AIC value due to the addition of a factor, respectively (Burnham and Anderson, 2002). Maximum likelihood (ML) estimation (Compound Symmetry models) were used to estimate the parameters of the models. However, an AIC value only compares one model to the next and does not indicate the absolute fit of the model to the data, therefore we also calculated the Akaike weight of each model (Burnham and Anderson, 2002). The Akaike weights compare all possible models and determine which model will come out best most of the time. Factors with non-significant main effects were excluded in the final models except when they were involved in significant higher interactions. We calculated Cohen’s *d* as a measure of effect size (<0.2—trivial, ≥ 0.2—small, ≥ 0.5—medium and ≥ 0.8—large). Significant findings from the models were explored using *post hoc* comparisons (paired *t*-test, two-tailed, Bonferroni adjusted for multiple comparisons). Lastly, we tested collinearity in the final models by determining the tolerance and variance inflation factors. A *p*-value of < 0.05 was considered significant for all statistical analyses. All values are expressed as the mean ± standard error of the mean (SEM).

## Results

### Baseline EEG

The analysis of the EEG data from the Pz electrode revealed that there was no significant difference between groups and stimulation conditions in the mean iAPF and mean posterior alpha power prior to the stimulation (all *p*s > 0.05; Table 1).

**Table 1:** Baseline measurements: individual alpha peak frequency (iAPF), alpha power, GMS and SS scores.

	Young group			Old group		
	Sham	α-tACS	α2-tACS	Sham	α-tACS	α2-tACS
iAPF	9.98 ± 0.11	10.05 ± 0.15	10.09 ± 0.07	9.98 ± 0.24	9.85 ± 0.14	10.01 ± 0.01
Alpha power	20.66 ± 2.95	18.58 ± 1.32	21.63 ± 1.86	22.14 ± 4.30	19.16 ± 1.57	21.50 ± 3.36
GMS score	0.032 ± 0.063	0.005 ± 0.054	0.015 ± 0.057	0.040 ± 0.115	0.003 ± 0.062	0.016 ± 0.074
SS score	0.110 ± 0.098	0.099 ± 0.106	0.116 ± 0.083	0.129 ± 0.094	0.081 ± 0.073	0.079 ± 0.084

*The mean iAPF (Hz), alpha power (dB), GMS score (ms), and SS score (ms) of the young and old groups across stimulation conditions before the stimulation. GMS, general motor skills; SS, sequence-specific motor skills; α-tACS, transcranial alternating current stimulation applied at iAPF; α2-tACS, transcranial alternating current stimulation applied at iAPF + 2 Hz*.

### Skill Acquisition Stage RT

In the first SRTT run, the mean RT for the sequential blocks gradually decreased for all participants. The RTs increased again in the second random block (block 6). The comparison of the calculated GMS scores (block 2–block 7) and SS scores (block 6–block 5) of the first SRTT run revealed no significant group or stimulation condition differences (all *p* = > 0.05; Table 1). Furthermore, no participant reported having been aware of the repeating sequence after the baseline SRTT measurement.

### TACS Stimulation and Skill Consolidation Stage RT

Overall, the participants tolerated the experimental procedure well. Except for four young participants who reported phosphene sensations (flickering light in the right visual field) only at the beginning of the α2-tACS stimulation, there were no reports of headaches, dizziness or nausea during and after stimulation. In all stimulation conditions, the averaged stimulation frequency did not differ significantly between the groups (Table 1). At the end of each experimental session, all participants noted the presence of repeating sequences but nobody could verbally recall the exact pattern.

In the final models, we excluded the GMS scores from 1,911 incorrect trials (5.06% of the total data) and 1,337 outlier RTs (3.54% of the total data). Similarly, we excluded the SS scores from 3,619 incorrect trials (7.18% of the total data) and 1,042 outlier RTs (2.07% of the total data). Therefore, the final model for the GMS score contained 91.41% of the total data set while the model for the SS score contained 90.75% of the total data set. In the final models for the GMS and SS scores, we included all the factors because a full model did come out best most of the time based on the Akaike weight (Supplementary Table S1). For the model of the GMS scores, even though the stimulation and group interaction effect was not significant (*F*_(2,20912.21)_ = 2.20, *p* = 0.111, *d* = 0.205), we included it in the final model because the individual main effects, as well as their higher interactions, were significant (Table 2). Similarly, the factor group (*F*_(1,35.00)_ = 0.89, *p* = 0.353, *d* = 0.158) was included in the final model for the SS scores because all of its interaction effects were significant. Moreover, the addition of these factors did not worsen the model fit based on the AIC values (Supplementary Table S1). All modeled data were normally distributed after logarithmic transformation (Shapiro–Wilk test) and the variances were equal for each group (Levene’s test; all *p* > 0.05). Tolerance range and variance inflation factors were equal to 1.000 in the final models indicating that multicollinearity had no effect on the findings. In addition, we are certain that results of the final models having a lower number of participants in the old group do not undermine the final results and interpretation because similar results were obtained when their data was modeled together with the young group that contained the same number of participants.

**Table 2:** Results of the linear mixed model (LMM) performed for the GMS and SS scores.

	Numerator *df*	Denominator *df*	*F*-value	*p-*value	Cohen’s *d*
**GMS score**				
Time	2	20,908.28	4.80	0.008*	0.151
Stimulation	2	20,912.12	6.52	0.001*	0.226
Group	1	35.01	8.85	0.005*	0.500
Time × stimulation	4	20,910.83	7.09	<0.001*	0.455
Time × group	2	20,908.28	12.47	<0.001*	0.586
Stimulation × group	2	20,912.12	2.20	0.111	0.205
Time × stimulation × group	4	20,910.83	5.99	<0.001*	0.478
**SS**				
Time	3	33,831.90	29.95	<0.001*	0.685
Stimulation	2	33,833.27	31.16	<0.001*	0.485
Group	1	35.00	0.89	0.353	0.158
Time × stimulation	6	33,831.97	3.98	0.001*	0.204
Time × group	3	33,831.90	3.42	0.017	0.177
Stimulation × group	2	33,833.27	54.82	<0.001*	0.302
Time × stimulation × group	6	33,831.97	5.24	<0.001*	0.263

For the LMM (random intercept model), each participant was treated as a random factor. The between-subjects factor group (young vs. old), and the within-subjects factors stimulation (sham and tACS) and time (prestimulation, 0 min, 60 min, and 120 min after stimulation) were treated as fixed factors. *significant main or interaction effect.

#### GMS Consolidation

**Figure 2:**
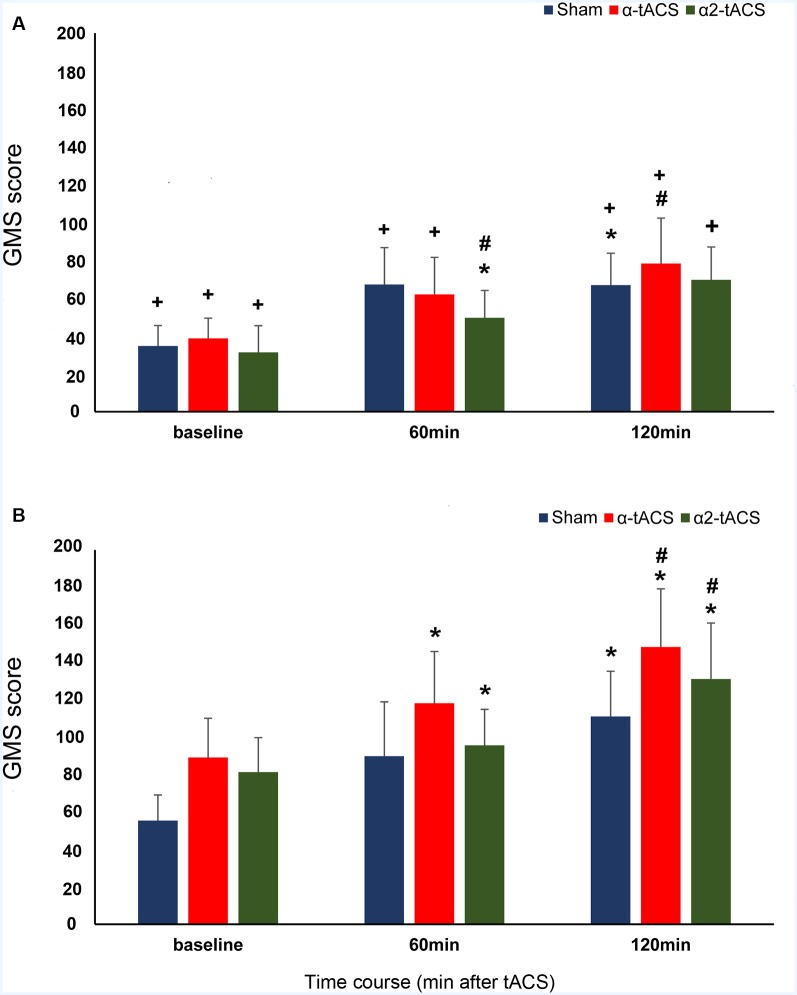
The effect of tACS stimulation on GMS consolidation. The y-axis displays the GMS scores in milliseconds (mean ± SEM). The GMS score is the difference in the reaction times (RT) from blocks with sequential trials (block 7 of the first SRTT run and block 2 of the following SRTT run). The x-axis displays the time points from which the GMS score was calculated. Baseline (RT difference between block 7 of the SRTT run before the stimulation and block 2 of the SRTT run immediately after stimulation), 60 min (RT difference between block 7 of the SRTT run immediately after stimulation and block 2 of the SRTT run 60 min after stimulation) and 120 min (RT difference between block 7 of the SRTT run 60 min after stimulation and block 2 of the SRTT run 120 min after stimulation). **(A)** Young group: α-tACS and α2-tACS stimulation had minimal impact on the GMS scores. **(B)** Old group: GMS scores significantly increased after α-tACS and α2-tACS stimulation and were significantly higher than sham 120 min after stimulation. The old group’s GMS scores were significantly higher than the young group’s for all time points except 60 min after α2-tACS stimulation. GMS, General motor skill; α-tACS, transcranial alternating current stimulation applied at iAPF; α2-tACS, transcranial alternating current stimulation applied at iAPF + 2 Hz, * = significant increase in GMS score compared to baseline, # = significant increase in GMS score compared to sham, + = significant difference between the young and the old group’s GMS score (Bonferroni corrected *post hoc* *t*-tests, paired, two-tailed, *p* < 0.05). Error bars denote SEM.

In our data, the RT difference between block 7 of the SRTT run before the stimulation and block 2 of the SRTT run immediately after stimulation served as the baseline GMS score. The results of the analysis revealed that the old group’s GMS scores were significantly higher than the young group’s after stimulation in all conditions except 60 min after α2-tACS stimulation (significant main effect of group: *F*_(1,35.01)_ = 8.85, *p* = 0.005, *d* = 0.500; significant time, stimulation and group interactions: *F*_(4,20910.83)_ = 5.99, *p* = <0.001, *d* = 0.478). In both groups, the GMS scores increased after stimulation (significant main effect of time: *F*_(2,20908.28)_ = 4.80, *p* = 0.008, *d* = 0.151). However, the changes in the GMS scores showed a group and a stimulation-specific effect (significant main effect of stimulation: *F*_(2,20912.12)_ = 6.52, *p* = 0.001, *d* = 0.226; significant time and stimulation interactions: *F*_(4,20910.83)_ = 7.09, *p* = <0.001, *d* = 0.455; significant time and group interactions: *F*_(2,20908.28)_ = 12.47, *p* = <0.001, *d* = 0.586; Figure 2). In the sham stimulation condition, the GMS scores increased and were significantly higher than the baseline 120 min after stimulation in both groups (young group: *p* = 0.036, old group: *p* = 0.005). The old group’s GMS scores were significantly higher compared to baseline 60 min (*p* = <0.001) and 120 min (*p* = <0.001) after α-tACS stimulation, as well as 60 min (*p* = 0.002) and 120 min (*p* = 0.042) after α2-tACS stimulation. In contrast, in the young group, the GMS score was only significantly higher than baseline 60 min (*p* = 0.028) after α2-tACS stimulation. When the real stimulation conditions were compared to sham, the results showed a significantly higher GMS score 120 min after α-tACS stimulation in both groups (young group—*p* = 0.030; old group—*p* = 0.003). On another hand, the GMS score was significantly higher than sham 120 min (*p* = <0.001) after α2-tACS stimulation in the old group, while the GMS score was significantly lower than sham (*p* = 0.031) 60 min after α2-tACS stimulation in the young group (Bonferroni corrected *post hoc* *t*-tests). In summary, α-tACS and α2-tACS improved GMS consolidation in the old group particularly 120 min after stimulation. In contrast, α-tACS had a minimal impact on GMS consolidation in the young group, while α2-tACS showed some detrimental effect on GMS consolidation 60 min after stimulation.

#### SS Consolidation

**Figure 3:**
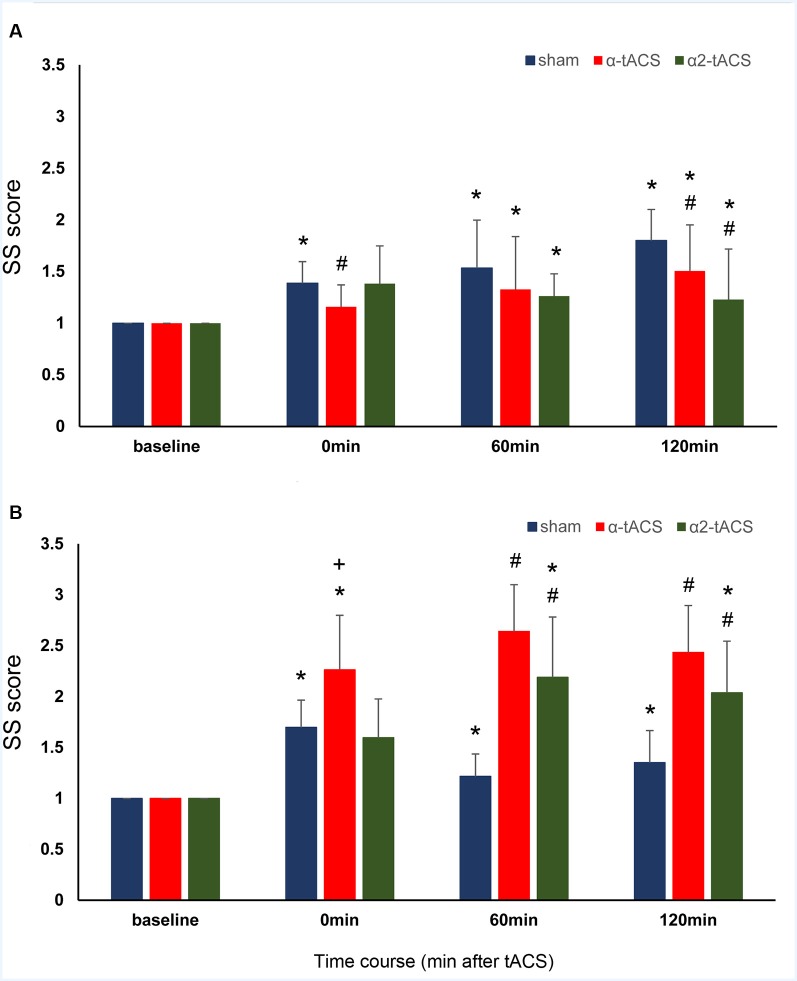
The effect of tACS stimulation on SS consolidation. The y-axis displays the SS scores (mean ± SEM) normalized with reference to the respective SS score before the stimulation (SS score after stimulation/SS score before stimulation). The SS score is the difference in the RT from the block with random trials (block 6) and a block with sequential trials (block 5) of the same SRTT run. The x-axis displays the time points before (baseline) and after stimulation (0 min or immediately after stimulation, 60 min and 120 min after stimulation). **(A)** Young group: SS scores were significantly lower than sham after α-tACS and α2-tACS stimulation. **(B)** Old group: SS scores significantly increased after α-tACS and α2-tACS stimulation and were significantly higher than sham 60 min and 120 min after stimulation. The old group’s SS scores were significantly higher than the young group’s immediately after α-tACS stimulation. SS, Sequence-specific skill; α-tACS, transcranial alternating current stimulation applied at iAPF; α2-tACS, transcranial alternating current stimulation applied at iAPF + 2 Hz, * = significant increase in SS score compared to baseline, # = significant increase in SS score compared to sham, + = significant difference between the young and the old group’s SS scores (Bonferroni corrected *post hoc* *t*-tests, paired, two-tailed, *p* < 0.05).

The analysis showed that SS scores changed significantly after the stimulation (significant main effect of time: *F*_(3,33831.90)_ = 29.95, *p* = <0.001, *d* = 0.685). The stimulation-induced changes in SS scores were found to be group-specific because the magnitude was higher in the old group compared to the young group (significant time and group interactions: *F*_(3,33831.90)_ = 3.42, *p* = 0.017, *d* = 0.177; Table 1 and Figure 3). A stimulation-specific effect can be observed on the SS scores as well (significant main effect of stimulation: *F*_(2,33833.27)_ = 31.16, *p* = <0.001, *d* = 0.485; significant time and stimulation interactions: *F*_(6,33831.97)_ = 3.98, *p* = 0.001, *d* = 0.204). In the young group, the SS scores were significantly lower immediately after (*p* = 0.006) and 120 min (*p* = <0.001) after α-tACS stimulation compared to sham at the same time points. In contrast, the SS scores were significantly higher than sham stimulation 60 (*p* = 0.001) and 120 min (*p* = <0.001) after α-tACS stimulation in the old group. Similarly, the SS scores were significantly lower than sham 120 min (*p* = 0.010) after α2-tACS stimulation in the young group, while the old group’s SS scores were significantly higher 60 min (*p* = <0.001) and 120 min (*p* = <0.001) after α2-tACS stimulation compared to sham (Bonferroni corrected *post hoc* *t*-tests). Moreover, although the old group exhibited overall higher SS scores than the young group after α-tACS and α2-tACS stimulation (significant stimulation and group interactions: *F*_(2,33833.27)_ = 54.82, *p* = <0.001, *d* = 0.302), the SS scores significantly differed between the groups only immediately after α-tACS stimulation (significant time, stimulation and group interactions: *F*_(6,33831.97)_ = 5.24, *p* = <0.001, *d* = 0.263). Here, the old group’s SS score was significantly higher (*p* = 0.046) than the young group’s SS score (Bonferroni corrected *post hoc* *t*-tests). In summary, the results showed better SS consolidation in the old group after α-tACS and α2-tACS stimulation compared to sham. On the other hand, both stimulation conditions had a detrimental effect on SS consolidation in the young group.

### ER

The overall ERs in the study were low. Calculated overall ER was 3.43% and 3.01% in the young and old group, respectively. Relative to the total number of trials in each stimulation condition, the young group only made errors in 3.34% of the trials in the sham condition, 3.11% of the trials in the α-tACS condition and 3.26% of the trials in the α2-tACS condition. In the old group, there were only errors in 2.81% of the trials in the sham condition, 2.92% of the trials in α-tACS condition, and 2.62% of the trials in the α2-tACS condition. The results of the initial full model indicated that ERs did not significantly differ across stimulation conditions and between the groups (Supplementary Table S2). However, the model showed a significant main effect of time and blocks, as well as the interaction effects of group and blocks. Therefore, we ran and interpret a reduced LMM that contained the factor group, block and time as well as their interactions. The final/reduced model showed similar results (Supplementary Table S2). The main effect of time was significant (*F*_(3,2869.06)_ = 17.74, *p* = <0.001, *d* = 0.353) indicating a significant increase in ER after stimulation. The main effect of block was also significant (*F*_(3,2869.06)_ = 17.74, *p* = <0.001, *d* = 0.705) indicating the significant difference in ER between sequential and random blocks. Furthermore, the *post hoc* comparisons for the significant group and block interactions (*F*_(6,2869.02)_ = 7.47, *p* = <0.001, *d* = 0.565) showed higher ERs in the young group (4.5%) compared with the old group (3.1%) only in block 1 (*p* = 0.026). Further comparisons revealed that these differences were also present during the consolidation stage (60 min: *p* = 0.048, 120 min: *p* = 0.029; Bonferroni corrected *post hoc* *t*-tests).

## Discussion

In the present study, we demonstrated the impact of tACS stimulation on motor skill consolidation in young and old participants. The EEG data analysis revealed comparable posterior alpha oscillatory activity in both groups. Although age-related changes in the posterior alpha rhythm such as slowing and reduction in peak power are typically reported in older adults, our participants in the old group may not yet have undergone these age-related changes. This is possible because the age of these participants was not advanced (mean age 61.66 ± 3.71 years), and they were physically active and very healthy. However, tACS stimulation applied at comparable alpha frequencies affected the two types of motor skill consolidation differently in both groups. In particular, α-tACS and α2-tACS improved GMS and SS consolidation in the old group. In contrast, α-tACS minimally improved GMS consolidation but impaired SS consolidation in the young group. Furthermore, α2-tACS was detrimental to the consolidation of both skills in the young group.

### The Acquisition of GMS and SS Skills Prior to the Stimulation

During the first SRTT run, the GMS score, SS score, and ER did not significantly differ between the groups indicating comparable skill learning. The participants also remained unaware of the test sequence, which is an indication that motor skill learning was largely implicit. The comparable group performance is not surprising because skill learning is generally preserved with age particularly when learning remains implicit and task complexity is low as in the SRTT task used here (Curran, 1997; Bennett et al., 2007; Brown et al., 2009; King et al., 2013; Meissner et al., 2016; Urry et al., 2018).

### Effect of tACS on GMS Consolidation

In SRTT, GMS consolidation specifically refers to performance improvement across blocks with the same movement sequence. Improved performance is indicated by the gradual increase in GMS scores which is due to the reduction of RTs because of the participant’s growing expertise on the movement component of the sequence. GMS consolidation is assumed to be accompanied by several processes such as stabilization and enhancement of motor memories, as well as resistance to interference (Meier and Cock, 2014). Sequential trials require a stable motor memory of sequential (repeating) finger movements. In our results, we observed an increase in GMS scores after each break indicating the improvement in GMS consolidation in both groups even in the absence of stimulation (sham condition). However, statistical comparisons of the group’s scores revealed that consolidation was significantly higher in magnitude in the old group compared to the young group. This could indicate that motor memories are either robust, less susceptible to interference or more efficiently retrieved in our old participants (Brashers-Krug et al., 1996). Nevertheless, the improved consolidation we observed in the old group was unexpected because studies generally report either reduced (relative to young subjects) or complete absence of performance improvement during retesting of motor sequence tasks in older adults (Brown et al., 2009; King et al., 2013; Cornelis et al., 2016; Meissner et al., 2016). Late improvement (after 12 or 24-h off-line period) of GMS was also reported in elderly individuals (Brown et al., 2009; Nemeth and Janacsek, 2011). In our study, one possible reason that could explain the better performance of the old group compared to the young group during the consolidation stage is the ceiling effect that could have prevented the young group in further improving their skill performance (Berghuis et al., 2015; Centeno et al., 2018). This is plausible because the young group’s scores tended to plateau whereas the old group’s score trend-wise increased after sham stimulation (Figure 2). A ceiling effect in performance is less observed in the elderly as they tend to show either smaller improvements during motor practice or require extended periods of training to achieve skill levels comparable to those obtained by younger adults (Roig et al., 2014). Therefore, the absence of a ceiling effect may give the old participants more room for improvement during re-testing.

In the real stimulation conditions, we observed a group and stimulation-specific effect on GMS consolidation scores. In both groups, the stimulation applied at iAPF (α-tACS) improved the consolidation as indicated by the approximately linear increase in GMS scores that became significantly higher than the effect of sham 120 min after stimulation. The facilitating effect of α-tACS on the young group’s motor skill performance fits well with the findings of previous studies that stimulated the left motor cortex with 10 Hz tACS. Young subjects showed improved sequence acquisition and early motor skill consolidation during stimulation (Antal et al., 2008; Pollok et al., 2015). On the other hand, the increase in consolidation we observed in our old group after α-tACS is a novel finding. In theory, α-tACS stimulation could potentially facilitate the consolidation process in both groups because the α-tACS stimulation frequency closely matched the spectral peak of ongoing motor-cortical alpha (10 Hz) activity (Ali et al., 2013). Another possibility to account for the smaller GMS scores in our young group compared to the old group is movement slowing in the young group. Movement slowing after 10 Hz tACS stimulation of the motor cortex was observed in a fast finger tapping task in young adults (Wach et al., 2013). Indeed, a small GMS score is indicative of a slower response in block 2.

The group-specific effect on GMS consolidation was also evident after α2-tACS stimulation. The young group’s GMS scores were significantly lower compared to sham while the old group’s GMS scores were significantly higher compared to sham 60 and 120 min after stimulation, respectively. These results suggest that stimulation above the endogenous oscillatory frequency was detrimental to young participants but beneficial for older participants. Although post-stimulation EEG measurements were not conducted, which can be considered a potential limitation of the present study, the detrimental effect of α2-tACS stimulation in the young group might be explained by the results of entrainment studies. Cortical oscillations were shown to be strongly suppressed for stimulation frequencies between the dominant endogenous frequency and its first harmonic frequency (Ali et al., 2013). Also, the strongest entrainment is expected in areas that show a preference for the entraining frequency (Ruhnau et al., 2016). Neuronal entrainment is less likely in the α2-tACS condition because motor-cortical alpha rhythm in the motor cortex oscillates at 10 Hz and our stimulation frequency is more or less 12 Hz (iAPF + 2 Hz). Even if α2-tACS stimulation could entrain the natural frequency in the motor cortex to a value higher than its natural value, it could still be detrimental because the increased oscillatory activity might lead to a higher than necessary and therefore disruptive motoneuronal activity in the young group (Feurra et al., 2011a; Wach et al., 2013). On the other hand, although the magnitude was less compared to the effect of α-tACS, GMS consolidation in the old group also improved after α2-tACS stimulation. This might be explained by the finding that a mismatched stimulation frequency can succeed in increasing the endogenous oscillation (Schmidt et al., 2014). However, why did mismatched stimulation work for the old group but not for the young group? This might be due to differences between young and older adults in brain oscillations during motor processing. In a study by Quandt et al. (2016), young participants exhibited a clear and peaked modulation of movement-related power decrease in the alpha and upper beta band during a finger sequence task, whereas there was a more uniform flat curve of alpha power decrease in older adults.

### Effect of tACS on SS Consolidation

In contrast to GMS consolidation, SS consolidation refers to performance improvement across sequenced and random trials. Specifically, the SS score refers to the increase in response times (RTs) when a random block directly followed a sequential block. Here, the resistance to the interfering memories of sequential finger movements might be the predominant reason for consolidation because the participants must inhibit the continued performance of the learned sequence. Indeed, compared to intelligence-matched controls, a core deficit in response inhibition is associated with slower RT’s especially in random trials in an SRTT task among the carrier of fragile X mental retardation 1 (FMR1) gene (Kraan et al., 2014). The lower accuracy in SRTT (including random trials) of individuals with attention deficit hyperactivity disorder (ADHD) is also ascribed to their impaired response inhibition (Pedersen and Ohrmann, 2018).

Similar to GMS consolidation, group and stimulation-specific effects on SS consolidation were evident in the two real tACS stimulation conditions. For instance, the young group’s SS scores after α-tACS were lower than those in the sham stimulation condition. In contrast, the old group’s SS scores were significantly higher after α-tACS than after sham stimulation for all time points. The group differences reached significance immediately after α-tACS stimulation. These results suggest that α-tACS stimulation is beneficial for the old group but detrimental for the young group. We would argue that the different effect of α-tACS on SS consolidation in both groups was due to the impact of stimulation on motor cortical inhibitory networks. In elderly adults, the inhibitory control of learned and automated motor response is suggested to be impaired due to age-dependent deterioration of motor cortical inhibitory networks. This deterioration is suggested to be reflected in EEG oscillatory changes over the sensorimotor regions particularly the motor cortical alpha power which is thought to be an index of inhibitory control (Hummel et al., 2002, 2004; Klimesch et al., 2007; Jensen and Mazaheri, 2010; Sauseng et al., 2013; Bönstrup et al., 2015). For instance, there is an impaired rebound of alpha power increase in the sensorimotor cortex after motor sequence learning and a reduced ability to inhibit inappropriate responses in older adults (Vallesi et al., 2010; King et al., 2013; Bönstrup et al., 2015; Mary et al., 2015). Interestingly, focal increases of oscillatory alpha activity were also absent over the primary motor cortex in dystonic patients who were known to have a deficit of inhibitory motor control (Hummel et al., 2002). Despite the absence of post-stimulation EEG measurement in our study, we may speculate that motor-cortical alpha power and inhibitory control is reduced in our old group compared to the young group after stimulation. Therefore, α-tACS-induced motor-cortical alpha power increase (through entrainment) will be more robust in elderly individuals because of low oscillatory power that could provide a bigger window of modulation. Behaviorally, the stimulation may increase inhibitory control crucial for tasks such as the SRTT because motor memories of sequential finger movements must be inhibited when performing random trials. Young adults typically exhibit a significant alpha power increase at the sensorimotor cortices during the consolidation of a skill that requires inhibition of the learned movements (Zhuang et al., 1997; Bönstrup et al., 2015). However, α-tACS have been shown to decrease cortical inhibition in young adults and therefore may cancel out the task-induced increase in inhibition (Wach et al., 2013; Fresnoza et al., 2018). If this is true, performance in blocks with random trials should be affected. Indeed, significantly higher ERs in block 1 were observed in the young group compared to the old group after α-tACS stimulation. The high ER could have been due to inefficient motor inhibition that leads to interference on the restabilization of task rule memory at the start of each SRTT run. Impaired inhibition was indicated by the low SS scores after α-tACS stimulation.

In the α2-tACS condition, although the magnitude is different, the direction of the stimulation after-effect on SS consolidation resembles that of α-tACS. α2-tACS stimulation of the motor cortex impaired and improved consolidation compared to sham in the young and old group, respectively. We believe that the same line of argumentation we presented for GMS consolidation can be applied to these results. For instance, in the young group, an increase in motor-cortical alpha power is not expected in the α2-tACS condition due to mismatched stimulation frequency (Ali et al., 2013; Ruhnau et al., 2016). This can negatively impact SS consolidation because of deficient cortical inhibition. On the other hand, improved consolidation after α2-tACS stimulation is possible in the old group because the mismatched stimulation frequency has the potential to increase alpha power in this age group (Schmidt et al., 2014). However, as our data showed, SS consolidation improvement was less in magnitude after α2-tACS compared to α-tACS in the old group. We argue that α2-tACS stimulation may induce an excessive increase in motor-cortical alpha power in the old group which by itself can be also detrimental to SS consolidation because of excessive inhibition. For example, very high prestimulus motor-cortical alpha activity could make the motor system less responsive to inputs from other brain regions (e.g., PFC) even for signals serving to inhibit an automatic motor action (Mazaheri et al., 2009). Higher intracortical inhibition in the older adults after α2-tACS could also reduce task-dependent plasticity which is crucial for motor skill consolidation (Mary et al., 2015).

## Conclusion

Our results show that motor skill consolidation can be enhanced with tACS stimulation of the motor cortex in elderly individuals. On the other hand, for young adults, tACS was detrimental to motor skill consolidation. The overall results of the present study suggest that the effects of tACS are age-dependent, that is, they depend on the overall level of alpha activity in the individual (Neuling et al., 2013). Our results suggest that tACS stimulation can remedy impaired oscillatory brain activity in order to improve motor and cognitive functions in the elderly, similar to earlier studies (Borghini et al., 2018). However, in determining the optimal stimulation frequency, it is important to individually identify the endogenous (dominant) resonant frequency for the underlying neuronal processes associated with the targeted brain area. In the primary motor cortex, 10 Hz tACS stimulation may increase “motor surround inhibition,” which is necessary for the selective activation of target muscles (e.g., the finger that should move) and the inhibition of nearby muscles (e.g., those fingers that should not move). This is necessary to produce the desired movements and prevent those unwanted (Naro et al., 2017). However, there are exceptions. For example, while cerebellar Purkinje cells’ average spike frequency is 10 Hz (Llinás, 2009), tACS applied at 10 Hz to the cerebellum was ineffective in modulating motor cortex excitability and motor performance probably because it had no or only a minimal effect on another type of inhibition called “cerebellum-brain inhibition” (CBI), as compared to 50 Hz tACS (Naro et al., 2017). In rabbits, motor behavioral effects (eyeblink) were induced at higher frequencies (30, 100 and 200 Hz) rather than 10 Hz (Márquez-Ruiz et al., 2016). Similarly, although tACS stimulation of the somatosensory cortex (SI) at alpha and high-gamma frequency was able to elicit tactile sensations (Feurra et al., 2011b), 10 Hz tACS did not modulate the ability to temporally discriminate between two subsequent tactile suprathreshold stimuli (Wittenberg et al., 2019). Overall, our data suggest that perturbations of neural oscillations can be beneficial for individuals with aberrant oscillations such as the elderly. On the other hand, for young healthy individuals with functional neural oscillation, minimal beneficial effects might be outweighed by detrimental effects.

## Data Availability Statement

The datasets generated for this study are available on request to the corresponding author.

## Ethics Statement

The studies involving human participants were reviewed and approved by Medical University of Graz. The patients/participants provided their written informed consent to participate in this study.

## Author Contributions

SF, MC, EG, CK, UZ, and AI designed the study. SF, MC, and LB conducted the experiments and collected the data. SF, LB, and AI analyzed the data and wrote the manuscript. SF, MC, LB, EG, CK, UZ, and AI reviewed and approved the final version of the manuscript.

## Conflict of Interest

The authors declare that the research was conducted in the absence of any commercial or financial relationships that could be construed as a potential conflict of interest.
