# Supplementary material for: Age-Dependent Effect of Transcranial Alternating Current Stimulation on Motor Skill Consolidation
Source: Front Aging Neurosci. 2020 Feb 6;12:25. doi: 10.3389/fnagi.2020.00025 (PMC7016219; doi:10.3389/fnagi.2020.00025)
Supplement: Supplementary file 1 [file Table_1.DOCX]

| **Model** | **Parameters** | **AICᵢ** | **Δᵢ (AIC)** | Relative likelihoods | ***w*ᵢ (AIC)** |
| --- | --- | --- | --- | --- | --- |
| **GMS** | Random intercept | 31337.980 | 75.985 | 0.000 | 0.000 |
|  | + Time | 31334.111 | 72.116 | 0.000 | 0.000 |
|  | + Stimulation | 31325.295 | 63.3 | 0.000 | 0.000 |
|  | + Time x stimulation | 31305.395 | 43.4 | 0.000 | 0.000 |
|  | + Group | 31299.481 | 37.486 | 0.000 | 0.000 |
|  | + Group x time | 31278.556 | 16.561 | 0.000 | 0.000 |
|  | + Group x stimulation | 31277.929 | 15.934 | 0.001 | 0.000 |
|  | + Group x stimulation x time | 31261.995 | 0 | 1.000 | 0.999 |
|  |  |  |  | Sum = 1.001 |  |
|  |  |  |  |  |  |
| **SS** | Random intercept | 36449.645 | 271.475 | 0.000 | 0.000 |
|  | + Time | 36352.897 | 174.727 | 0.000 | 0.000 |
|  | + Stimulation | 36316.218 | 138.048 | 0.000 | 0.000 |
|  | + Time x stimulation | 36306.840 | 128.67 | 0.000 | 0.000 |
|  | + Group | 36307.949 | 129.779 | 0.000 | 0.000 |
|  | + Group x time | 36303.720 | 125.55 | 0.000 | 0.000 |
|  | + Group x stimulation | 36197.598 | 19.428 | 0.000 | 0.000 |
|  | + Group x stimulation x time | 36178.170 | 0 | 1.000 | 1.000 |
|  |  |  |  | Sum = 1.000 |  |

**Supplementary Table 1. Akaike weights of GMS and SS score models.** First, we identified the model with the lowest AIC value (AICᵢ). Second, we calculated the AIC difference (Δᵢ (AIC) by subtracting each AIC to the AICᵢ. The AIC difference was then used to calculate the relative likelihood needed for calculating the Akaike weights (wᵢ (AIC)). The model with the biggest wᵢ (AIC) is the best model.
