# Supplementary material for: Age-Dependent Effect of Transcranial Alternating Current Stimulation on Motor Skill Consolidation
Source: Front Aging Neurosci. 2020 Feb 6;12:25. doi: 10.3389/fnagi.2020.00025 (PMC7016219; doi:10.3389/fnagi.2020.00025)
Supplement: Supplementary file 2 [file Table_2.DOCX]

|  | Numerator df | Denominator df | *F*-value | *p-*value | Cohen’s d |
| --- | --- | --- | --- | --- | --- |
| **Full Model** |  |  |  |  |  |
| Group | 1 | 35.01 | .55 | .463 | 0.173 |
| Stimulation | 2 | 2481.97 | 1.63 | .196 | 0.058 |
| Time | 3 | 2481.35 | 13.05 | <.001 | 0.353 |
| Blocks | 6 | 2481.29 | 61.03 | <.001 | 0.705 |
| Group x stimulation | 2 | 2481.97 | 2.89 | .056 | 0.173 |
| Group x time | 3 | 2481.35 | .66 | .576 | 0.130 |
| Group x blocks | 6 | 2481.29 | 4.26 | <.001 | 0.565 |
| Stimulation x time | 6 | 2481.30 | .87 | .514 | 0.260 |
| Stimulation x blocks | 12 | 2481.26 | .21 | .998 | 0.026 |
| Times x blocks | 18 | 2481.26 | 1.06 | .388 | 0.108 |
| Group x stimulation x time | 6 | 2481.30 | .65 | .688 | 0.130 |
| Group x stimulation x blocks | 12 | 2481.26 | 1.19 | .285 | 0.391 |
| Group x time x blocks | 18 | 2481.27 | .33 | .996 | 0.241 |
| Stimulation x time x blocks | 36 | 2481.27 | .62 | .996 | 0.103 |
| Group x stimulation x time x blocks | 36 | 2481.27 | .50 | .995 | 0.142 |
| **Reduced Model** |  |  |  |  |  |
| Group | 1 | 34.90 | .49 | .489 | 0.173 |
| Time | 3 | 2869.06 | 17.74 | <.001 | 0.353 |
| Blocks | 6 | 2869.02 | 76.77 | <.001 | 0.705 |
| Time x blocks | 18 | 2869.02 | 1.60 | .052 | 0.294 |
| Group x time | 3 | 2869.06 | .13 | .945 | 0.130 |
| Group x block | 6 | 2869.02 | 7.47 | <.001 | 0.565 |
| Group x blocks x time | 18 | 2869.02 | .24 | 1.000 | 0.241 |

**Supplementary Table 2. Results of the linear mixed model (LMM) performed for the error rates.** For the LMM (random intercept model), each participant was treated as a random factor. In the full model, the between-subjects factor group (young vs old), as well as the within-subjects factors stimulation (sham, α-tACS, and α2-tACS), time (prestimulation, 0min, 60 min, and 120min after stimulation) and blocks (block 1 to 7) were treated as fixed factors. In the reduced model, the fixed factor stimulation was excluded.
